# Novel signals for naive lymphocyte recruitment under inflammation

**DOI:** 10.1186/s43556-025-00270-x

**Published:** 2025-05-13

**Authors:** Weilong Zheng, Chunsheng Dong, Fangfang Zhou

**Affiliations:** 1 The First Affiliated Hospital, the Institutes of Biology and Medical Sciences, Suzhou Medical College, Soochow University, Suzhou, 215123 China; 2 Institutes of Biology and Medical Sciences, Soochow University, 199 Ren-Ai Road, Suzhou Industrial Park, Suzhou, Jiangsu Province 215123 China

A recent study published [1] in *Cell* by Chen et al. revealed key chemotactic signals driving lymphocyte recruitment to inflamed lymph nodes (LNs): the CC chemokine receptor 7 (CCR7) undergoes dynamic ligand switching, with CC chemokine ligand 19 (CCL19) superseding CCL21 to guide naïve lymphocyte homing. In addition, EBI2 (GPR183) and its oxysterol ligand 7α,25-dihydroxycholesterol (7α,25-HC) provide indispensable chemoattractant signals for lymphocyte recruitment during inflammation (Fig. 1) [1].

**Fig. 1:**
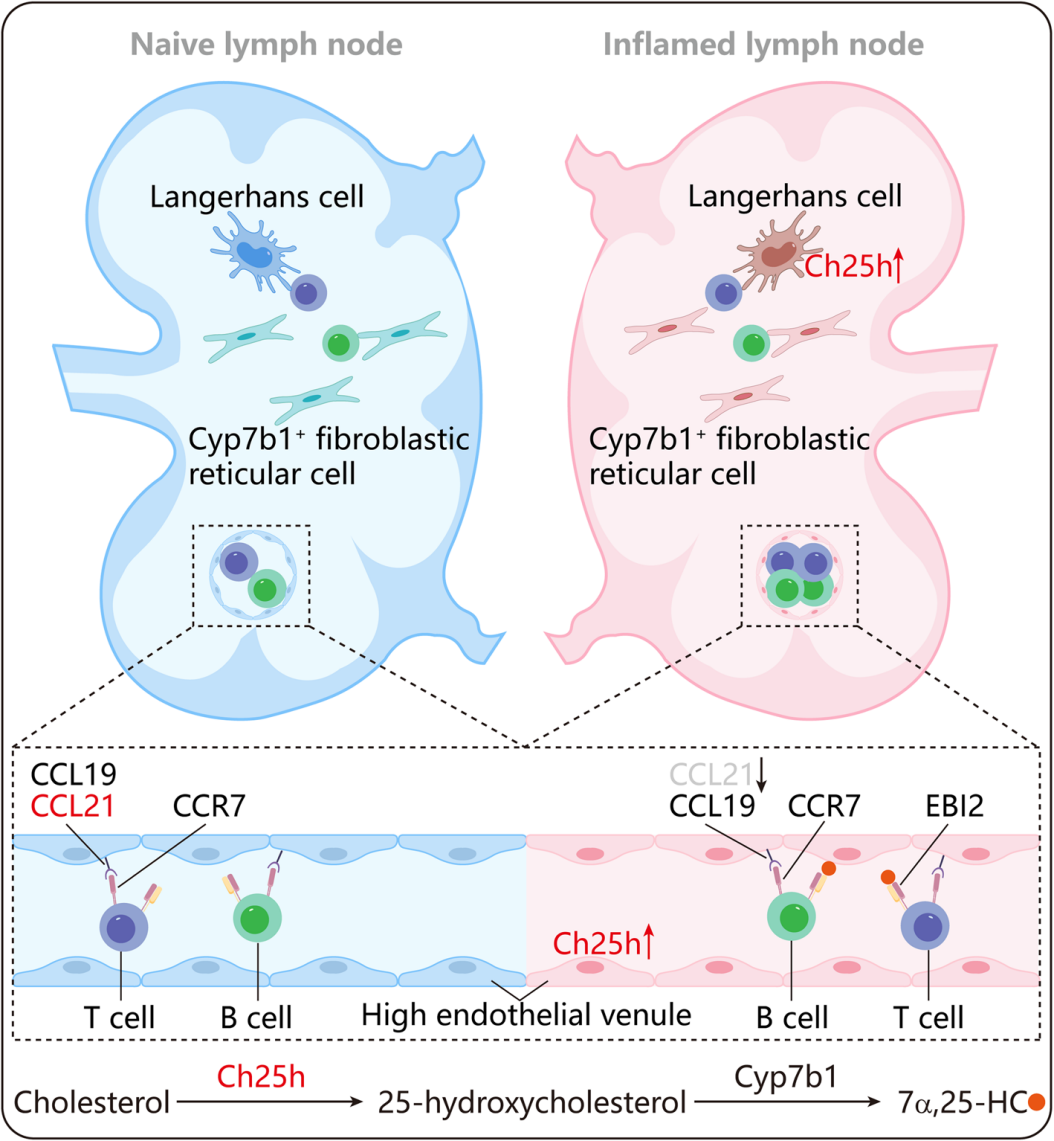
Schematic representation of lymphocyte recruitment dynamics in lymph nodes under homeostatic and inflammatory states. Inflammation suppresses CCL21, shifting the CCR7 ligand for lymphocyte recruitment from CCL21 to CCL19 and increasing reliance on the EBI2-oxysterol ligand 7α,25-HC signal

Previous studies have demonstrated significantly downregulation of CCL21 in LNs following immunization or infection or in those draining certain tumors [2, 3]. Notably, mice deficient in CCL19 exhibit normal lymphocyte homing and LN architecture under steady-state conditions [4]. Based on these observations, this study explores whether alternative signals, such as the EBI2-oxysterol ligand axis, supports the recruitment of naïve lymphocyte to inflamed LNs during CCL21 downregulation [1].

Cholesterol 25-Hydroxylase (Ch25h) and 25/26-Hydroxycholesterol 7-Alpha-Hydroxylase (Cyp7b1) orchestrate the enzymatic cascade producing 7α,25-dihydroxycholesterol (7α,25-HC), the endogenous oxysterol ligand activating the EBI2 chemotactic receptor. EBI2 exhibits robust expression in naïve B cells, mediating their follicular homing within lymphoid tissues, but displays markedly reduced abundance across CD4 and CD8 T cells [5]. Chen et al. reported that EBI2-deficient B cells exhibited impaired inflamed LN entry, with this functional defect being dependent on the EBI2 oxysterol ligand 7α,25-HC. The expression of Ch25h is enriched in both murine and human high endothelial venules (HEVs). Following lymphocytic choriomeningitis virus (LCMV) challenge, Ch25h undergoes significant upregulation not only in LN vasculature but also within interfollicular and outer T-zone stromal cells at 24-h post-infection. Intriguingly, the induction of Ch25h temporally coincides with a pronounced suppression of CCL21 chemokine expression within the same microenvironment. Unlike Ch25h, Cyp7b1 is widely expressed in fibroblastic reticular cells (FRCs) within the T-cell zone of LNs, showing no significant alterations at 24-h post-infection [1]. To investigate the relationship between the 7α,25-HC: EBI2 axis and inflamed LNs entry. The authors conducted a mixed adoptive transfer experiment during LCMV infection using mice with *Ch25h*, *Cyp7b1,* and *EBI2* deletions. Deletion of these genes resulted in impaired recruitment of naïve B cells into inflamed LNs. Furthermore, EBI2-mediated entry into inflamed LNs was dependent on both Ch25h and Cyp7b1. While naïve CD4 and CD8 T cells required EBI2 signaling for LNs homing, this chemotactic dependence was less pronounced than in naive B cells, correlating with their lower receptor abundance. These findings indicate that the Ch25h-oxysterol-EBI2 axis may serve as a significant mediator of lymphocyte entry into the LNs during infection. Additionally, the authors reported that it specifically enhances the entry of B and CD8 T central memory cells into tumors, which is consistent with the high expression of EBI2 in these cell types.

Subsequently, the authors investigated specific Ch25h-derived cells that promote inflamed LN entry because the specific deletion of Ch25h in endothelial cells partially affected B cell recruitment into inflamed LN entry. Firstly, they performed a specific deletion of Ch25h in hematopoietic stem cells (Ch25h^Δhemat^) but observed a phenotype similar to that observed in Ch25h deletion experiments involving endothelial cells. Secondly, they conducted wild-type (WT) bone marrow (BM) transplantation into irradiated Ch25^Δhemat^ mice to evaluate the recovery of impaired inflamed LN entry. Transferring WT BM into Ch25^Δhemat^ chimeras resulted in little change in inflamed LNs compared to non-chimera Ch25^Δhemat^ mice. These findings indicate that the radioresistant hematopoietic cells exhibiting Vav-iCre activity contribute Ch25h to EBI2-dependent LN homing. Finally, the authors identified these cells as radioresistant Langerhans cells (LCs) and revealed significantly upregulated *Ch25h* expression in LCs within inflamed LNs, thereby contributing to oxysterol generation. In contrast, *Cyp7b1* was expressed at low levels in the LCs from both naïve and inflamed LNs. These results suggest that sustained inflamed LN entry mediated by oxysterol and EBI2 receptor-ligand signaling in the context of CXCL21 downregulation, results from the cooperation among endothelial cells, fibroblasts, and LCs.

Finally, while the expression of CCL19 remained stable in the inflamed LNs, the repression of CCL21 could affect the activity of CCL19 via reducing competitive binding. The author revealed the non-redundant roles of CCL19 and CCL21 in mediating lymphocyte migration into inflamed LNs and that blocking CCL19 amplified the lymphocyte homing defect caused by EBI2 deletion. Blocking both CCL19 and oxysterol nearly halts lymphocyte movement, highlighting their combined importance as critical pathways for lymphocyte entry into inflamed lymph nodes.

Chen et al. revealed the dynamic regulation of lymphocyte trafficking and a novel receptor-ligand system signal. For the first time, the authors introduced the coordinated production of oxysterols by multiple cells within the inflammatory milieu, which facilitates the recruitment of EBI2-expressing lymphocytes to lymph nodes and tumor sites. These findings support a model in which 7α,25-HC is synthesized stepwise, transcellularly. This mechanism involves the synthesis and secretion of 25-HC by HEVs, followed by its metabolism into 7α,25-HC by FRCs, which ultimately act on EBI2^+^ lymphocytes located near HEVs. Additionally, the authors revealed that the upregulation of Ch25h in LCs induced by inflammation also contributes to this signaling and promotes lymphocyte entry into inflamed LNs.

However, several questions must be addressed in future studies. For example, this study did not fully elucidate the mechanism underlying CCL21 downregulation. Although the authors suggested that the upregulation of Ch25h expression accompanies the downregulation of CCL21 during inflammation, the specific mechanism underlying Ch25h upregulation and the potential mutual inhibition between CCL21 and Ch25h have not been clarified. Additionally, while the authors investigated the expression of Ch25h in LCs and the effect of knocking out LCs on inflamed LN entry, they did not clarify whether the observed effects were due to the LCs themselves or to the impact of Ch25h expression on LCs. The production and effects of oxysterols are spatially distinct, and their dynamic transport and increased levels during inflammation may modulate other immune processes. Furthermore, the authors primarily focused on lymphocyte migration during inflammation but did not address how the dynamic regulation of lymphocyte trafficking affects the ability of adaptive immune cells.

In summary, these insights have significant potential for clinical applications, offering a deeper understanding of immune cell dynamics in inflammatory pathologies and paving the way for developing innovative therapeutic interventions. Modulation of the EBI2-oxysterol signaling axis and CCL19 represents a promising approach for precisely manipulating lymphocyte function in combating infections and tumors.

## Data Availability

Not applicable.
